# Factors influencing the uptake of voluntary HIV counseling and testing in rural Ethiopia: a cross sectional study

**DOI:** 10.1186/s12889-016-2918-z

**Published:** 2016-03-08

**Authors:** Hailay D. Teklehaimanot, Awash Teklehaimanot, Mekonnen Yohannes, Dawit Biratu

**Affiliations:** Center for National Health Development in Ethiopia, Columbia University, Kebele 06, H No 447, PO Box 664 code 1250, Bole Sub City, Addis Ababa Ethiopia; The Earth Institute, Columbia University, 475 Riverside Drive, Suite 401, New York, NY 10025 USA; College of Health Sciences, Mekelle University, PO Box 1871, Mekelle, Ethiopia

**Keywords:** HIV/AIDS, VCT utilization, HEP, HEWs, Model-family, Community conversation, Rural, Ethiopia

## Abstract

**Background:**

Voluntary counseling and testing (VCT) has been one of the key policy responses to the HIV/AIDS epidemic in Ethiopia. However, the utilization of VCT has been low in the rural areas of the country. Understanding factors influencing the utilization of VCT provides information for the design of context based appropriate strategies that aim to improve utilization. This study examined the effects of socio-demographic and behavioral factors, and health service characteristics on the uptake of VCT among rural adults in Ethiopian.

**Methods/design:**

This study was designed as a cross sectional study. Data from 11,919 adults (6278 women aged 15–49 years and 5641 men aged 15–59 years) residing in rural areas of Ethiopia who participated in a national health extension program evaluation were used for this study. The participants were selected from ten administrative regions using stratified multi-stage cluster sampling. Multivariate logistic regression analysis was performed accounting for factors associated with the use of VCT service.

**Results:**

Overall, men (28 %) were relatively more likely to get tested for HIV than women (23.7 %) through VCT. Rural men and women who were young and better educated, who perceived having small risk of HIV infection, who had comprehensive knowledge, no stigmatization attitude and discussed about HIV/AIDS with their partner, and model-family were more likely to undergone VCT. Regional state was also strongly associated with VCT utilization in both men and women. Rural women who belonged to households with higher socio-economic status, non-farming occupation, female-headed household and located near health facility, and who visited health extension workers and participated in community conversation were more likely to use VCT. Among men, agrarian lifestyle was associated with VCT use.

**Conclusions:**

Utilization of VCT in the rural communities is low, and socio-economic, behavioral and health service factors influence its utilization. For increasing the utilization of VCT service in rural areas, there is a need to target the less educated, women, poor and farming families with a focus on improving knowledge and reducing HIV/AIDS related stigma. Strategy should include promoting partner and community conversations, accelerating model-family training, and using alternative modes of testing.

## Background

Ethiopia, Africa’s second most populous nation, is among the most affected by the HIV epidemic in Sub-Saharan Africa, where unprotected heterosexual contact and mother-to-child transmission are the main routes of transmission [1, 2]. HIV/AIDS, which continues to be a major development challenge for Ethiopia with an estimated adult prevalence of 1.5 % in 2011, has stabilized in the past decade with heterogeneity between different geographic areas [3]. Although the HIV prevalence in rural Ethiopia was relatively low (0.9 %) [3], small market towns are becoming hotspots bridging the urban-to-rural spread of HIV [4]. Moreover, the rural population with disproportionately low access to HIV/AIDS services is vulnerable to the risk of HIV infection, and the further spread of HIV to rural population is likely to increase as the communication and transport infrastructure improves [5].

Voluntary counseling and testing (VCT) has been one of the key policy responses to the HIV/AIDS epidemic, principally as a primary prevention strategy and as an entry point to other HIV/AIDS related services [6]. While undergoing VCT services, individuals learn about their sero-status and gain knowledge on avoiding risky behaviors to protect themselves and others [7–9]. VCT also serves as the basis for accessing HIV treatment and care as well as emotional support that enable individuals to cope with HIV-related anxiety and plan for their future [7–9].

Despite its strategic importance, the VCT uptake has been low in Ethiopia, as elsewhere in Africa [3, 10–13]. In 2005, the VCT uptake in Ethiopia was extremely low with only 4 % of women and 6 % of men ever tested for HIV [11], which increased to 38.8 and 41.1 %, respectively in 2011 following the rapid scale up of primary health care facilities, under the rural Health Extension Program (HEP), and increased accessibility to free anti-retroviral drugs [3]. However, the VCT uptake in 2011 was lower among rural residents (30.9 % for women and 36 % for men) compared to the urban residents (63.8 % of women and 58.5 % of men).

It is widely accepted that the uptake of VCT is influenced by socio-demographic characteristics such as age, gender, marital status, educational attainment, socio-economic status, and area of residence [12–17], behavioral and psychosocial factors such as high risk sexual partner, HIV/AIDS related knowledge, confidentiality, self-perceived risk, stigma and discrimination, and perceived benefits of VCT [10, 12–22], and health service delivery environment [11, 14, 15, 23, 24].

Understanding the factors influencing the utilization of VCT provides information for the design of context based appropriate strategies to improve access. Nevertheless, studies undertaken to understand factors affecting VCT utilization among rural population of Ethiopia are limited [12, 25]. This study therefore seeks to examine the effects of socio-demographic and behavioral factors, and health service characteristics, with a focus on HEP, on the uptake of VCT among rural Ethiopian adults using a nationally representative sample.

## Methods

### Setting

The federal government structure of Ethiopia is composed of nine Regional States and two City Administrations (Addis Ababa and Dire-Dawa). The regions are Afar, Amhara, Benshangul-Gumuz, Oromia, Gambela, Harari, SNNP (Southern Nations and Nationalities and Peoples), Somali and Tigray. The regions are divided into woredas (districts), which are further divided into kebeles (the lowest administrative government units). Majority (83.6 %) of the people in Ethiopia reside in rural areas with about 10 % practicing pastoral and agro-pastoral livelihood. There are about 15,000 rural kebeles in the country, each with an average of 5000 inhabitants. The rural population has access to Primary Health Care Unit (PHCU), which is the lowest-tier in the healthcare delivery system comprising a cluster of five health posts and a referral health center. Over 15,000 health posts and 3000 health centers have been established to cover all rural areas in the country with PHCU. Over 38,000 high-school completed female Health Extension Workers (HEWs) who received one year training on HEP have been deployed to the health posts (at least two HEWs per health post). The candidates were recruited from their prospective villages to limit staff turnover and address gender, social and cultural factors in service provision. Volunteer community health workers (CHWs) who are trained as model family support the community level activities of HEWs.

The HEP package comprises 16 services covering family health, communicable diseases, and sanitation and hygiene programs. With the aims to develop personal and social skills, and increase health awareness that enable individuals to promote their own health, HEP focuses on promotive and preventive interventions with limited curative services. In addition to the promotive and preventive HIV/AIDS services, HEWs who are deployed in high HIV prevalent areas also provide HIV counseling and testing (HCT). The referral health centers are staffed with higher health professionals and all provide HCT services.

### Study design and sampling methods

This study is part of a comprehensive HEP evaluation survey conducted in 2010 with the aim to assess the implementation process and effect of HEP on health outcomes of the rural population. The evaluation was designed as a multi-level cross-sectional study involving household, health worker, and health facility surveys. The data for this article came from the household survey.

The households were sampled from rural kebeles using a stratified multi-stage cluster sampling method with region as strata, and district, kebele and household as primary, secondary, and tertiary sampling units, respectively. The sampling frame comprised 679 districts with 14,723 rural kebeles from the 10 regions (including the rural areas of Dire Dawa). The sampling procedure involved selection of 71 districts and 312 kebeles through systematic-random sampling with probability-proportional-to-size. A cross-sectional sample of 7128 households was selected from the 312 kebeles using the random-walk method used in the Expanded Program of Immunization cluster surveys. Household level data was collected using five modules: Socio-demographic (20 items), Family Health (90 items), Malaria and Tuberculosis (69 items), HIV/AIDS (57 items), and Hygiene and Sanitation (34 items), which were developed with some modification to the Ethiopian Demographic and Health Survey (EDHS) questionnaires [11]. The module on household socio-demographic characteristics was administered to the heads of 7098 households. The HIV/AIDS module was administered to 12,060 adults (women aged 15–49 years and men aged 15–59 years).

### Consent and confidentiality of study data

The study has received approval from the national ethics review committee of the Ethiopian Federal Ministry of Science and Technology, and the institutional review board of the Columbia University (IRB-AAAC8935). Study subjects were recruited in person by survey supervisors. The need for written informed consent was waived, and oral consent was obtained from each respondent using an approved information statement. Strict confidentiality of information and anonymity of data have been kept throughout the study. Data collection was carried out in January and February of 2010.

### Measurements

For the analysis reported in this paper, we used data from 11,919 adults (6278 women and 5641 men) with matching data on socio-demographic and HIV/AIDS. The dependent variable was VCT utilization. The independent variables included socio-demographic, behavioral and programmatic factors. The socio-demographic variables were: gender, age, marital status, educational level, primary occupation of household head, region of residence, settlement type (pastoralist/agro-pastoral and agrarian), household’s socioeconomic status and religion. Socio-economic status (SES) index variable was constructed for each household from a set of self-reported assets and living condition variables that reflect economic status using principal component analysis and households were grouped into five clusters using k-means clustering in STATA/IC 12.0 [26].

The programmatic variables included walking distance to the nearest health facility (<=10, 10–30 and >30 min), contact with HEWs (proactively visiting HEWs and visited by HEW at home), exposure to HIV/AIDS related information (never exposed, exposed through mass media, and exposed through community conversation), model-family and availability of volunteer CHWs in the village. HIV/AIDS related communications involving health workers, HEWs, community volunteers, community members and school were all considered as community conversation activities.

The behavioral variables included high-risk sexual partner in the past 12 months, self-perceived risk of HIV infection (four categories: no, small, moderate/great risk and don’t know), belief that HIV/AIDS is fatal, belief that HIV/AIDS can be cured, HIV/AIDs knowledge index and stigma scale, and conversation with partner about HIV. Knowledge index was constructed by combining the responses to 7 sets of questions: a healthy-looking person can have the HIV virus; HIV can’t be transmitted by mosquito bites, supernatural means and sharing food; and HIV can be prevented through abstinence, being faithful to one uninfected partner and condom use. The scores were categorized into four groups: none (0–1), low (2–3), moderate (4) and high (5–7). The stigma index was constructed using four standard questions: willingness to care if a relative becomes ill with HIV; willingness to buy fresh vegetable from a food seller who has the HIV virus; allowing child to play with a child who has HIV virus; and keeping secret about a family member infected with HIV virus. The scores were categorized into four groups: no (0–1), low (2), moderate (3) and high (4) stigma.

### Statistical analysis

Descriptive statistics were generated to characterize the study subjects. Univariate analysis was employed to independently assess the statistical association of the independent factors and the dependent variable. All socio-demographic variables were entered into a multivariate logistic model, while other variables were considered based on cut-off p-value (<0.1) after testing multicollinarity between all variables. The analysis was carried out for men and women separately in STATA version 12, and survey weights were specified to account for sampling design in generating results that included odds ratios (OR) and 95 % confidence intervals (CI).

## Results

### Characteristics of the study population

A total of 11,919 people were included in the analysis (Table 1). The median age was 28 years for women (52.7 %) and 33 years for men (47.3 %). Majority of respondents came from male-headed (85 %) and farming households (90 %). Majority (78.9 %) were married and about 58 % (70.9 % of women and 47.1 % of men) had never attended or completed one year of formal education. About 14.5 % of respondents were from pastoral or agro-pastoral communities.

**Table 1:** Background characteristics of study population by gender, rural Ethiopia, 2010

		Total		Women		Men	
Variables		N	%	N	%	N	%
Socio-demographic variables							
Overall		11,919		6278	52.7	5641	47.3
Age group, year							
	15–19	1342	11.3	770	12.3	572	10.1
	20–24	1785	15.0	1123	17.9	662	11.7
	25–29	2196	18.4	1357	21.6	839	14.9
	30–39	3635	30.5	1921	30.6	1714	30.4
	40+	2961	24.8	1107	17.6	1854	32.9
Marital status							
	Married	9400	78.9	4973	79.3	4427	78.5
	Never married	1860	15.6	758	12.1	1102	19.6
	Divorced/Widowed	651	5.5	542	8.6	109	1.9
Educational level							
	Never attended/<1 year	6934	58.2	4386	70.9	2548	47.1
	Primary	2835	23.8	1183	19.1	1652	30.5
	Secondary or higher	1832	15.4	619	10.0	1213	22.4
Gender of household head							
	Female	1794	15.1	1233	19.7	561	10.0
	Male	10,113	84.8	5041	80.4	5072	90.0
Occupation of household head							
	Farmer	10,688	89.7	5599	90.3	5089	91.3
	Gov't employee/merchant	569	4.8	314	5.1	255	4.6
	Other	519	4.4	291	4.7	228	4.1
Religion							
	Orthodox	4749	39.8	2493	39.8	2256	40.1
	Islam	4122	34.6	2170	34.6	1952	34.7
	Protestant	2527	21.2	1331	21.3	1196	21.3
	Other	494	4.1	270	4.3	224	4.0
Socio-economic status index							
	Low	2084	17.5	1123	17.9	961	17.1
	Low-middle	4192	35.2	2233	35.6	1959	34.8
	Middle	3586	30.1	1863	29.7	1723	30.6
	High-middle	1720	14.4	884	14.1	836	14.8
	High	329	2.8	170	2.7	159	2.8
Settlement pattern							
	Pastoral/agro-pastoral	1724	14.5	909	14.5	815	14.5
	Agrarian	10,195	85.5	5369	85.5	4826	85.6
Region							
	Tigray	1152	9.7	622	9.9	530	9.4
	Afar	358	3.0	191	3.0	167	3.0
	Amhara	2677	22.5	1409	22.4	1268	22.5
	Oromia	2852	23.9	1461	23.3	1391	24.7
	Benshangul-Gumuz	644	5.4	330	5.3	314	5.6
	SNNP	2063	17.3	1052	16.8	1011	17.9
	Gambela	1128	9.5	649	10.3	479	8.5
	Dire Dawa	137	1.1	83	1.3	54	1.0
	Harari	170	1.4	93	1.5	77	1.4
	Somali	738	6.2	388	6.2	350	6.2
Behavioral variables							
Risk partner in past 12 months							
	No	11,694	98.1	6177	98.4	5517	97.8
	Yes	225	1.9	101	1.6	124	2.2
Self-perceived risk of HIV							
	No risk	7128	59.8	3581	57.0	3547	62.9
	Small risk	1183	9.9	573	9.1	610	10.8
	Moderate/great risk	708	5.9	360	5.7	348	6.2
	Don’t know	2900	24.3	1764	28.1	1136	20.1
Believes HIV/AIDS is fatal							
	No	2113	17.7	1336	21.3	777	13.8
	Yes	9806	82.3	4942	78.7	4864	86.2
Believes HIV/AIDS can be cured							
	No	10,321	86.6	5458	86.9	4863	86.2
	Yes	1598	13.4	820	13.1	778	13.8
HIV/AIDS knowledge index							
	None	4151	34.8	2597	41.4	1554	27.6
	Low	2592	21.7	1328	21.2	1264	22.4
	Moderate	3007	25.2	1455	23.2	1552	27.5
	High	2169	18.2	898	14.3	1271	22.5
HIV/AIDS stigma scale							
	No stigma	4222	35.4	1907	30.4	2315	41.0
	Low stigma	2049	17.2	1020	16.3	1029	18.2
	Moderate stigma	3009	25.2	1671	26.6	1338	23.7
	High stigma	2639	22.1	1680	26.8	959	17.0
Talked with partner about HIV							
	No	6436	54.0	3668	58.4	2768	49.1
	Yes	5483	46.0	2610	41.6	2873	50.9
Programmatic variables							
Walking distance to HF							
	<=10 min	9510	79.8	5016	79.9	4494	79.7
	10–30 min	1639	13.8	861	13.7	778	13.8
	30+ minutes	770	6.5	401	6.4	369	6.5
Proactively visited HEW							
	No	7242	60.8	3839	62.5	3403	62.2
	Yes	4373	36.7	2304	37.5	2069	37.8
HEW visited home							
	No	6511	54.6	3474	57.0	3037	55.9
	Yes	5016	42.1	2624	43.0	2392	44.1
Source of HIV information							
	Never exposed	1207	10.1	833	13.3	374	6.6
	Only to mass media	745	6.3	338	5.4	407	7.2
	Community conversations	9967	83.6	5107	81.4	4860	86.2
Model-family							
	No	11,221	94.1	5917	95.4	5304	95.1
	Yes	555	4.7	283	4.6	272	4.9
VHPs in village							
	No	5594	46.9	2995	47.7	2599	46.1
	Yes	6325	53.1	3283	52.3	3042	53.9

About 2 % of respondents reported high-risk sexual partner in the 12 months prior to the survey, and majority (59.8 %) perceived no risk of HIV infection, while only 5.9 % perceived moderate or great risk. Only 18.2 % (14.3 % of women and 22.5 % of men) had comprehensive knowledge about HIV/AIDS. While 35.4 % (30.4 % of women and 41 % of men) had no stigma towards people living with HIV, about 22 % (26.8 % of women and 17 % of men) had high levels of stigma.

About 80 % of respondents reside within 10 min walking distance to health facility. About 37 % proactively visited HEWs during the month prior to the survey. Majority (83.6 %) was exposed to HIV/AIDS related information through community conversations. Overall, 25.8 % (23.7 % for women and 28 % for men) had been tested for HIV. Over half (53.1 %) of respondents were from villages with active CHWs, and only 4.7 % were from model-family households.

### Univariate logistic regression

The result of univariate logistic regression analysis by gender is presented in Table 2. Socio-demographic factors significantly associated with VCT uptake among both women and men were: age and educational attainment, occupation of household head, wealth index, agro-ecological zone, and region. The results showed that self-perceived risk of HIV, belief on whether HIV/AIDS is fatal, and conversation with partner about HIV/AIDS were significantly associated with VCT uptake in both men and women. VCT uptake increased significantly with increased levels of HIV/AIDS related knowledge index, while it decreased significantly with increasing stigma scale. Risky sexual partner in the past 12 months was significantly associated with VCT uptake only among women. Contact with HEWs, sources of HIV information, model-family, and CHWs were significantly associated with the use of VCT in both genders. However, proximity to a health facility was only significantly associated with VCT uptake among women.

**Table 2:** Result of logistic regression of the association between VCT uptake and explanatory factors by gender, rural Ethiopia 2010

		Women				Men			
Variables		Unadjusted		Adjusted		Unadjusted		Adjusted	
		OR	95 % CI	OR	95 % CI	OR	95 % CI	OR	95 % CI
Socio-demographic variables									
Age group, year									
	15–19	1		1		1		1	
	20–24	1.43	1.13–1.80	1.43	1.06–1.92	1.93	1.51–2.47	1.68	1.19–2.37
	25–29	1.07	0.80–1.44	1.13	0.73–1.76	1.99	1.50–2.63	1.84	1.26–2.68
	30–39	0.85	0.65–1.11	0.85	0.57–1.27	1.51	1.16–1.96	1.33	0.86–2.06
	40+	0.56	0.42–0.73	0.66	0.45–0.96	1.09	0.82–1.46	1.08	0.67–1.72
Marital status									
	Married	1		1		1		1	
	Never married	1.3	0.99–1.71	0.69	0.47–1.02	0.95	0.78–1.17	0.76	0.54–1.09
	Divorced/Widowed	1.38	1.01–1.90	1.11	0.71–1.75	0.86	0.42–1.76	1.03	0.47–2.26
Educational level									
	Never attended/<1 year	1		1		1		1	
	Primary	2.41	1.90–3.06	1.71	1.29–2.26	2.04	1.56–2.68	1.68	1.35–2.09
	Secondary and higher	3.1	2.28–4.20	1.66	1.21–2.28	3.12	2.36–4.13	2.37	1.79–3.14
Gender of household head									
	Female	1		1		1		1	
	Male	0.9	0.62–1.30	0.53	0.39–0.73	1.77	1.14–2.75	1.11	0.80–1.54
Occupation of household head									
	Farmer	1		1		1		1	
	Gov't employee/merchant	2.58	1.79–3.70	1.65	1.13–2.41	1.95	1.46–2.61	1.41	0.89–2.24
	Other	1.02	0.60–1.72	1.29	0.64–2.59	0.66	0.39–1.11	1.06	0.53–2.12
Religion									
	Orthodox	1		1		1		1	
	Islam	0.88	0.54–1.43	1.84	1.22–2.78	0.8	0.48–1.32	1.56	1.06–2.31
	Protestant	1.21	0.77–1.89	0.98	0.61–1.57	1.45	0.99–2.12	1.27	0.78–2.06
	Other	0.9	0.48–1.67	1.37	0.64–2.94	0.79	0.46–1.34	1.27	0.49–3.28
Socio-economic status index									
	Low	1		1		1		1	
	Low-middle	1.44	1.04–2.00	1.24	0.94–1.64	1.52	1.07–2.16	1.21	0.88–1.66
	Middle	2.01	1.35–3.00	1.30	0.93–1.80	1.77	1.21–2.60	1.01	0.72–1.43
	High–middle	2.46	1.63–3.74	1.43	1.03–1.98	2.4	1.60–3.60	1.11	0.75–1.64
	High	4.48	2.53–7.94	1.86	1.12–3.09	3.36	1.99–5.68	1.3	0.73–2.29
Settlement pattern									
	Pastoral	1		1		1		1	
	Agrarian	2.02	1.04–3.90	1.4	0.78–2.50	3.08	1.79–5.30	1.92	1.15–3.20
Region									
	Tigray	1		1		1		1	
	Afar	0.35	0.18–0.67	0.38	0.14–1.00	0.31	0.18–0.56	0.43	0.16–1.2
	Amhara	0.42	0.22–0.82	0.69	0.41–1.16	0.56	0.29–1.1	0.84	0.46–1.54
	Oromia	0.26	0.14–0.47	0.34	0.16–0.74	0.36	0.19–0.68	0.4	0.18–0.85
	Benshangul-Gumuz	0.31	0.12–0.82	0.53	0.21–1.33	0.37	0.17–0.81	0.44	0.15–1.27
	SNNP	0.57	0.31–1.06	0.74	0.36–1.53	0.78	0.45–1.35	0.73	0.37–1.47
	Gambela	0.19	0.09–0.36	0.36	0.16–0.83	0.18	0.09–0.38	0.21	0.08–0.54
	Dire Dawa	0.75	0.17–3.26	1.81	0.46–7.10	0.67	0.41–1.11	1.92	0.82–4.52
	Harari	0.19	0.11–0.35	0.50	0.23–1.10	0.14	0.09–0.24	0.2	0.1–0.42
	Somali	0.02	0.01–0.07	0.03	0.01–0.11	0.03	0.01–0.08	0.08	0.03–0.21
Behavioral variables									
Risk partner in past 12 months									
	No	1		1		1		1	
	Yes	1.93	1.16–3.21	1.91	0.79–4.64	1.86	0.95–3.66	1.91	0.63–5.83
Self perceived risk of HIV									
	No risk	1		1		1		1	
	Small risk	1.99	1.29–3.06	2.34	1.35–4.07	1.46	0.94–2.25	1.86	1.15–3.02
	Moderate/great risk	0.73	0.42–1.26	0.95	0.60–1.51	1.01	0.59–1.74	1.26	0.86–1.84
	Don’t know	0.25	0.17–0.36	0.73	0.50–1.09	0.27	0.19–0.40	0.62	0.42–0.90
Believes HIV/AIDS is fatal									
	No	1		1		1		1	
	Yes	3.71	2.58–5.34	0.88	0.61–1.28	3.52	2.50–5.00	1.27	0.89–1.82
Believes HIV/AIDS can be cured									
	No	1				1			
	Yes	1.25	0.91–1.73			0.94	0.69–1.27		
HIV/AIDS knowledge index									
	None	1		1		1		1	
	Low	3.45	2.43–4.91	1.50	1.06–2.13	2.71	1.86–3.93	1.50	1.05–2.15
	Moderate	4.51	3.12–6.51	1.57	1.11–2.23	3.71	2.45–5.63	1.56	1.05–2.33
	Comprehensive knowledge	8.39	5.37–13.11	2.32	1.45–3.72	5.98	3.85–9.30	1.96	1.23–3.13
HIV/AIDS stigma scale									
	No stigma	1		1		1		1	
	Low stigma	0.68	0.51–0.90	0.84	0.65–1.09	0.59	0.47–0.74	0.70	0.56–0.87
	Moderate stigma	0.42	0.32–0.56	0.67	0.49–0.92	0.38	0.30–0.49	0.58	0.42–0.80
	High stigma	0.22	0.14–0.72	0.65	0.46–0.93	0.25	0.17–0.36	0.56	0.38–0.80
Talked with partner about HIV									
	No	1		1		1		1	
	Yes	4.5	3.58–5.7	2.27	1.86–2.77	3.93	2.94–5.25	2.09	1.61–2.71
Programmatic variables									
Walking distance to HF									
	<=10 min	1		1		1		1	
	10–30 min	0.55	0.37–0.81	0.76	0.60–0.97	0.76	0.57–1.02	0.80	0.64–1.00
	30+ minutes	0.45	0.27–0.76	0.60	0.42–0.87	0.62	0.36–1.05	0.76	0.56–1.03
Proactively visited HEW									
	No	1		1		1		1	
	Yes	2.16	1.74–2.69	1.40	1.13–1.73	1.86	>1.5–2.30	1.23	099–1.52
HEW visited home									
	No	1		1		1		1	
	Yes	2.16	1.66–2.81	1.22	0.97–1.54	2	1.55–2.60	1.22	0.98–1.53
Exposure to HIV information									
	Never exposed	1		1		1		1	
	Only to mass media	8.31	3.34–20.71	5.42	1.89–15.54	6.07	2.08–17.76	1.91	0.69–5.29
	Community conversations	19.68	8.61–45–02	5.92	2.38–14.72	12	4.55–31.59	2.52	0.96–6.65
Household graduated as model-family									
	No	1		1		1		1	
	Yes	3.06	2.09–4.48	1.50	1.03–2.18	4.34	2.81–6.70	2.65	1.72–4.07
VHPs in village									
	No	1		1		1		1	
	Yes	3.12	2.14–4.54	1.44	1.00–2.06	2.91	2.02–4.19	1.37	0.98–1.90

### Multivariate logistic regression

#### Women

The result of multivariable logistic regression by gender is presented in Table 2. Socio-demographic factors independently associated with increased VCT uptake among women were: 20–24 years age group (OR = 1.4; 95 % CI 1.1–1.9); attainment of primary (OR = 1.7; 95 % CI 1.3–2.3) and secondary/higher (OR = 1.7; 95 % CI 1.2–2.3) education; household head being merchant or government employee (OR = 1.7; 95 % CI 1.2–2.8); Islam religion (OR = 1.8; 95 % CI 1.2–2.8); and wealth index – middle-high (OR = 1.4; 95 % CI 1.03–2.0) and high (OR = 1.9; 95 % CI 1.1–3.1); while male-headed household was associated with decreased uptake of VCT (OR = 0.5; 95 % CI 0.4–0.7). Women’s marital status and agro-ecological settlement were not significantly associated with VCT uptake.

Women who perceived themselves to be at small risk of HIV were 2.3 times more likely to receive VCT in comparison with those who perceived to have no risk. The odds of VCT uptake among women with comprehensive HIV/AIDS knowledge were 2.3 (95 % CI 1.45–3.72) times higher than those who lacked any HIV/AIDS related knowledge. The uptake of VCT was higher among women who talked about HIV with their partner (OR = 2.27; 95 % CI 1.86–2.77). The uptake of VCT among women with high levels of stigma was significantly lower (OR = 0.65; 95 % CI 0.46–0.93) in comparison with those who showed positive attitude.

Women who walk for more than 30 min to reach the nearest health facility were less likely (OR = 0.60; 95 % CI 0.42–0.87) to receive VCT in comparison with those who reside within 10 min walking distance. The odds of VCT uptake among women who proactively visit HEWs were 1.40 (95 % CI 1.13–1.73) times higher than among those women who did not visit HEWs. The uptake of VCT was higher among women who received HIV information through community conversations (OR = 5.92; 95 % CI 2.38–14.72) in comparison with those who were not exposed to any HIV related information. The odds of VCT uptake among women from model-family were 1.50 (95 % CI 1.03–2.18) times higher than among those from non-model family. Likewise, the odds of VCT uptake among women from villages with active CHWs were 1.44 (95 % CI 1.00–2.06) times higher than among those from villages without CHWs.

#### Men

Socio-demographic factors independently associated with increased VCT uptake among men were: 20–29 years age group (20–24 years: OR = 1.7; 95 % CI 1.2–2.4 and 25–29 years: OR = 1.8; 95 % CI 1.3–2.7); attainment of primary (OR = 1.7; 95 % CI 1.4–2.1) and secondary/higher (OR = 2.4; 95 % CI 1.8–3.1) education; Islam religion (OR = 1.6; 95 % CI 1.1–2.3); and agrarian (settled) dwellers (OR = 1.9; 95 % CI 1.2–3.2). Marital status, household head’s gender, occupation and wealth index were not significantly associated with VCT uptake among men respondents.

Men who perceived themselves to be at small risk of HIV were 1.86 (95 % CI 1.15–3.02) times more likely to receive VCT in comparison with those who perceived to have no risk; while men who could not state their perceived risk of HIV were less likely be tested (OR = 0.62; 95 % CI 0.42-0.90) compared to those with no risk. The odds of VCT uptake among men with comprehensive HIV/AIDS knowledge were 1.96 (95 % CI 1.23–3.13) times higher than among those men who lacked any HIV/AIDS related knowledge. The uptake of VCT was higher among men who talked with their partner about HIV (OR = 2.09; 95 % CI 1.61–2.71). The uptake of VCT among men with high levels of stigma towards people living with HIV was significantly lower (OR = 0.56; 95 % CI 0.38–0.80) in comparison with those who showed positive attitude.

The odds of VCT uptake among men from model-family were 2.65 (95 % CI 1.72–4.07) times higher than among their counter parts. All other programmatic variables included in this study were not significant predicators of VCT uptake among men.

## Discussion

While the utilization of VCT improved considerably compared to 2005 levels, which is likely due to the increased number of health facilities providing HCT services, introduction of lay counselors and provider-initiated testing initiatives, and increased accessibility to free anti-retroviral drugs [3, 11], the uptake in rural areas was low in comparison with the urban areas [3]. This study examined the association between socio-demographic, behavioral and health service factors and VCT uptake in rural Ethiopia using a nationally representative cross sectional sample of adults. The findings in this study has shown that several socio-demographic, behavioral and health service related factors were associated with HIV testing among rural men and women.

The findings showed that the proportions ever tested were significantly higher among youth, which is somehow encouraging as youth are at particularly higher risk of HIV acquisition. Our findings agree with studies conducted in Ethiopia [3], and elsewhere in Africa [14, 19, 27].

Consistent with the national level EDHS data and other studies, the results show better-educated individuals are more likely to be tested for HIV [3, 16, 19, 28, 29]. Women from households with greater economic wealth were more likely to be tested for HIV consistent with other studies [3, 11]. Education and wealth are positively related to certain risk factors such as premarital sex [30], and associated with higher HIV prevalence [3]. Related with the effect of socio-economic status, women belonging to households whose primary occupation was trade and government job were more likely to use VCT [31].

The results showed significant difference in the uptake of VCT by region, which partly reflect the multicultural characteristics of the country and difference in the pace of implementation of the HEP. The percentage that has ever been tested for HIV ranged from about 2 % in Somali to nearly 50 % in Tigray. The lowest percentages of HIV testing were observed in regions with predominantly pastoral lifestyle, consistent with the overall poor health indicators reported for these regions [32]. The lower uptake of VCT in Gambella is critical as Gambela is one of the regions with the highest level of HIV prevalence (6.5 %) in the country [3]. The remote and harsh climatic condition and the highly mobile and geographically dispersed settlement coupled with poor infrastructure and social services could have contributed to the observed low uptake of VCT. However, the findings of our study suggest that agro-ecological zone had varied influence on VCT uptake in men and women. While belonging to an agrarian community was a strong predictor of VCT uptake among men, the association was non-significant among women. The overall low uptake of VCT among women regardless of agro-ecological zone suggests that all rural women face similar socio-cultural barriers on VCT utilization [20]. Respondents belonging to Muslim religion were more likely to be tested, which is in contrast to other studies that reported either lack of association [33], or the opposite findings [12].

With regard to behavioral factors, VCT uptake increased sharply with increased HIV/AIDS related knowledge, which is consistent with other studies [16, 34]. The observed strong inverse association between stigma and VCT uptake is consistent with other studies, and in light of the high levels of stigma, the findings suggest that fear of stigma and discrimination is an important barrier to HIV testing [10, 12, 13, 19, 35]. The result showed that respondents with a small-perceived risk of HIV were more likely to use VCT compared to those with no-perceived risk of HIV. The low uptake of VCT among those who perceived no risk of infection, which could be due to lack of motivation to undergo HIV testing, has negative effect on the overall VCT uptake as this perception was common (60 %). In contrast to rural Tanzania [17], but consistent with findings from rural Uganda and Southern African countries [36–38], there was no evidence to suggest that a high-perceived risk of HIV was independently associated with VCT uptake. A study among youth in Ethiopia reported high-perceived risk of infection was associated with low VCT uptake [39], which could be due to fear of discrimination, in light of the prevalent stigma [22]. Individuals who talked with their partners about HIV/AIDS were more likely to use VCT, likely due to its positive effect on stigma and discrimination.

While all the programmatic factors affected VCT uptake among women, model-family was the only factor that affected VCT uptake among men. Women who resided further away from health facility were less likely to undergone VCT, likely due to household responsibilities limiting their time to travel long distance to seek health services. Women who were exposed to HIV-related information through community conversation were 5.9 times more likely to use VCT in comparison with women who were never exposed to such information. It has been reported that exposure to community conversation, particularly when individuals who were tested speak openly about HIV, led to greater acceptance and uptake of testing [37, 40, 41], which could be due to peer pressure [42]. The observed influence of volunteer CHWs and contact with HEWs in the uptake of VCT among women may be related to increased frequency of conversation about HIV. With regards to contact with HEWs, while proactive contact was associated with HIV testing, being visited at home by HEWs did not have noteworthy effect on HIV testing, which suggests that motivation play an important role, over and above the mere exposure to health information, in influencing HIV testing behaviors. On the other hand, the lack of influence of community conversation, CHWs and contact with HEWs on VCT uptake among men, and the strong influence of model-family on VCT uptake among men suggests that it takes intensive effort to bring about meaningful behavioral change among men. To graduate as a model-family, households undergo intensive theoretical and practical training on the various HEP services including HIV/AIDS for about 96 h, and are required to implement and adopt the HEP package [4].

In the present study, men were relatively more likely to get tested than women, which is consistent with EDHS data [3], and other studies from rural Uganda [43], South Africa [44], and Zimbabwe [16]. Socio-economic status and distance to health facility, which are important barriers to VCT use among women, could have contributed to the lower uptake among women [38]. Moreover, gender inequality and socio-cultural barriers could affect VCT demand among women [20]. In Ethiopia, fear of abandonment and divorce following discovery of HIV positive status were some of the barriers reported by women for freely seeking VCT services [22]. Although not all studies confirm gender differences in general attitudes toward testing [19]; women’s decisions about testing could be affected by their childbearing and breastfeeding plans, and their husband’s opinion [45–47]. Our finding, on the other hand, contrast with reports of higher testing rates among women from a number of other studies [25, 31, 37, 48–50], which could be due to more frequent interaction with health services during antenatal care and prevention of mother to child transmission programs [40, 41]. However, the observed disparity could be largely due to differences in the study design and study population (rural vs urban; national vs localized samples).

### Limitations

The study has some limitations. Due to the cross-sectional design of the current study, causal inferences cannot be drawn from the associations. Since data was collected in face-to- face interviews, information bias might occur due to recall, sensitivity of questions, and social desirability factors. Information bias could have contributed to the low percentage (<2 %) of respondents who reported high-risk sexual behavior, which might have resulted in the lack of an association with HIV testing. Similarly, HIV testing status may be subjected to information bias. To minimize such bias, interviewers were trained to ask questions in a non-judgmental way, and respondents were assured that personal information would be confidential and de-identifiable. Moreover, engagement in high-risk sexual practice and undergoing HIV testing are easily remembered, and thus recall bias may not be a major challenge. Despite these limitations, the use of a large representative population-based sample allowing for greater generalizability and the examination of a wide range of explanatory factors were the main strengths of the current study.

## Conclusion

Findings from the current study have important implications for developing intervention programs targeting rural areas in Ethiopia. First, the findings highlight individual- and community-level characteristics that influence the utilization of HIV testing, and the need for more intense efforts targeting the less educated, poor and farming families [51]. Second, the variation across regions and agro-ecological zones requires targeted capacity building and adopting appropriate delivery modalities such as mobile VCT services for pastoral regions. Third, promoting partner and community conversation, which has been the core government intervention in the national HIV/AIDS strategy [4], and speeding the scale-up of the model-family implementation should be given utmost priority to address the HIV related low knowledge and prevalent stigma. The recently introduced women development army network, which links five households to one model family, will be an important platform for effective community conversation and peer education [4]. Finally, making VCT a routine service in all health posts and provision of home-based VCT as well as provider initiated modes of testing would address the physical access barrier to rural women, while also tackling demand side barriers such as stigma and confidentiality [51–53].

## Availability of data and materials

The dataset supporting the conclusions of this article is available in the Open Science Framework repository (https://osf.io/7hqz8/files/).

## Abbreviations

CHWs: community health workers
EDHS: Ethiopian Demographic and Health Survey
HCT: HIV counseling and testing
HEP: health extension program
HEW: health extension worker
HIV/AIDS: Human Immunodeficiency Virus/Acquired Immunodeficiency Disease Syndrome
PHCU: Primary Health Care Unit
PMTCT: prevention of mother to child transmission
SES: socio-economic status
SNNP: Southern Nations and Nationalities and People
VCT: voluntary counseling and testing
